# Distribution of *Leptospira interrogans* by Multispacer Sequence Typing in Urban Norway Rats (*Rattus norvegicus*): A Survey in France in 2011-2013

**DOI:** 10.1371/journal.pone.0139604

**Published:** 2015-10-08

**Authors:** Florence Ayral, Anne-Laure Zilber, Dominique J. Bicout, Angeli Kodjo, Marc Artois, Zoheira Djelouadji

**Affiliations:** 1 WildTech, USC 1233, Université de Lyon-VetAgro Sup, Marcy L’Etoile, France; 2 USC 1233, Université de Lyon-VetAgro Sup, Marcy L’Etoile, France; 3 USC 1233, Laboratoire des Leptospires, Université de Lyon-VetAgro Sup, Marcy L’Etoile, France; 4 Biomatématiques et Epidémiologie, EPSP-TIMC, UMR CNRS 5525, Université Grenoble-Alpes, VetAgro Sup, Marcy L’Etoile, France; University of Kentucky College of Medicine, UNITED STATES

## Abstract

**Background:**

Urban leptospirosis has increasingly been reported in both developing and developed countries. The control of the disease is limited because our understanding of basic aspects of the epidemiology, including the transmission routes of leptospires among rat populations, remains incomplete. Through the ability to distinguish among *Leptospira* strains in rats, multispacer sequence typing (MST) could provide a modern understanding of *Leptospira* epidemiology; however, to our knowledge, the distribution of *Leptospira* strains among urban rat colonies has not been investigated using MST.

**Aims and Methodology:**

The objective of this study was to identify the *Leptospira* strains present in rats (*Rattus norvegicus*) in Lyon (France) using MST and to characterize their spatial distribution. Kidneys and urine were collected from rats trapped live in seven locations in the city and in one suburban location. Each location was considered to represent a rat colony. Bacterial cultures and quantitative polymerase chain reaction (qPCR) assays were performed, and the *L*. *interrogans* DNA identified was then genotyped using MST. The distributions of *Leptospira* strains were spatially described.

**Key Results:**

Among 84 wild rats, MST profiles were obtained in 35 of 37 rats that had a positive result for *L*. *interrogans* by bacterial culture and/or qPCR analyses. All of the MST profiles were related to reference strains previously isolated from human patients that belong to the serogroup Icterohaemorrhagiae and the serovars [strain(s)] Copenhageni [Wijinberg or M20] (n = 26), Icterohaemorrhagiae [CHU Réunion] (n = 7), Icterohaemorrhagiae [R1] (n = 1) and Copenhageni [Shibaura 9] (n = 1). Each colony was infected with leptospires having the same MST profile.

**Major Conclusions:**

This study demonstrated that MST could be used for the purpose of field studies, either on culture isolates or on DNA extracted from kidneys and urine, to distinguish among *L*. *interrogans* isolates in rats. MST could thus be used to monitor their distributions in urban rats from the same city, thereby providing new knowledge that could be applied to explore the circulation of *L*. *interrogans* infection in rat colonies. Because the strains are related to those previously found in humans, this application of MST could aid in the source tracking of human leptospirosis, and the findings would be relevant for public health purposes according to the One Health principle.

## Introduction

It has been estimated that more than one million severe cases of leptospirosis occur per year worldwide [1], and leptospirosis is considered to be an emerging disease in many countries [2]. The infection can cause fever, renal failure and pulmonary hemorrhage and is potentially fatal in 5% to 15% of cases [3–5]. It is a bacterial zoonosis caused by pathogenic *Leptospira*, resulting from direct or indirect contact with the urine or tissues of infected animals [6]. Although many animals can be carriers, rats are the most common source of human infection, particularly in urban environments, as rats are a synanthropic species (adapted to coexisting with the human population) [7–9]. Environmental factors also play a role in leptospirosis, leading to a higher incidence in tropical countries; however, because rats are ubiquitous, leptospirosis remains a public health issue even in cities of developing countries [8–10].

Pathogenic species include *L*. *interrogans*, *L*. *kirschneri*, *L*. *noguchii*, *L*. *borgpetersenii*, *L*. *weilii*, *L*. *santarosai*, *L*. *alexanderi*, *L*. *alstonii* and *L*. *mayottensis* [11–13]. *Leptospira* can also be classified into approximately 300 serovars, based on the lipopolysaccharide (LPS) structure. Antigenically related serovars have been grouped into 25 serogroups [11]. Because the species identification is not related to the LPS-associated genes (*i*.*e*., O-antigen gene cluster), the molecular and the serology-based taxonomies do not entirely overlap [14]. Different serovars are adapted to different mammalian hosts, which can either act as a reservoir of a co-adapted *Leptospira* serovar or carry and disseminate other serovars [6]. It is therefore difficult to track strains with molecular identities or serotypes that change with the hosts and the environments they inhabit or pass through.

Rats are colonial and territorial [15] animals that promote intra-colony interactions. In the urban environment, limited available space leads to the formation of colonies with a small home range, and inter-colony contacts may occur. Therefore, the urban rat population more likely functions as a meta-population, as described by Viana *et al*. [16], in which the sub-unit is the rat colony. Determining rat meta-population behavior with regard to *Leptospira* strain distribution would provide evidence of rat interactions resulting in rat infections. These investigations are of public health importance according to the One Health principle, as they could aid in understanding of the spatial distribution of infected rats, leading to potential disease transmission to humans. A molecular tool that can identify and monitor the isolates circulating in urban rat colonies is required for the investigation of potential routes of contamination (intra *vs*. inter colony) and their relative importance.

To successfully establish the epidemiological relationship between any two isolates, it is necessary to type them at a sufficient level of resolution [17]. Rats have been reported as the primary carriers of *Leptospira* serovars Icterohaemorrhagiae and Copenhageni (*i*.*e*., the Icterohaemorrhagiae serogroup) [8;18]. Therefore, the molecular data on the leptospires circulating in urban rat populations at the serovar level are not sufficient, and opportunities to distinguish at the isolate level could provide new perspectives for epidemiological analysis and for source tracking, in particular. Other than serological methods, molecular tools that have been employed to detect and genotype leptospiral agents in rat surveys include pulse field gel electrophoresis (PFGE), analyses of variable number tandem repeats (VNTR), and multi-locus sequence typing (MLST) [19–22]. However, these methods suffer from low discriminatory power and can only distinguish between *Leptospira* species, serogroups or serovars. O-genotyping could be a relevant method for identifying isolates; however, because of a lack of sequences of O-antigen gene clusters from various serogroups, this method has not been developed in *Leptospira*. Recently, multispacer sequence typing (MST) technology has been used and validated to distinguish between *L*. *interrogans* at the strain level in both human and animal samples. Because it is based on sequencing, MST has particularly high discriminatory power for the Icterohaemorrhagiae serogroup compared with VNTR [23]. The method can generate sequence data that are suitable for epidemiological and rat population studies. To our knowledge, no previous studies have applied MST for the purpose of a survey of wild rats.

The objectives of the study were as follows: (1) to identify the MST profile of *L*. *interrogans* isolates circulating in rat colonies in central Lyon, France, and in suburbs of Lyon; and (2) to characterize the distribution of MST profiles in rat colonies over space. Based on the results of this study, the use of MST for the surveillance of *Leptospira* in animal hosts should be considered, and we offer a public health perspective on the potential for its use.

## Materials and Methods

### Ethics statement

The authors assert that all procedures conducted in this study complied with the ethical standards of the European regulation governing the care and use of laboratory animals, following the agreement n° 69–020931, delivered by Rhône Préfecture (S1 File).

### Study area

The study was conducted in Lyon (N45°45’/E4°50’) and included seven trapping locations within the city center and one in a suburb. The eight urban trapping locations encompassed five dwelling areas: “Mermoz-B” N45°43’43.16”/E4°53’09.63”, “Mermoz-F” N45°43’38.47”/E4°53’05.35”, and “Mermoz-R” N45°43’44.96/E4°52’57.56” were from an inner-city neighborhood; “Guillotière” N45°45’08.45”/E4°50’08.45” included ancient buildings; and “Chapelle” N45°45’52.58/E4°47’24.28” included more recent buildings, a public garden (“Parc de la Tête d’Or” N45°46’26.93”/E4°44’04.06”), the waste incineration plant (“UIOM” N45°43’26.95”/E4°50’16.75”) and a grassland in the suburb (“Francheville” N45°44’28.87/E 4°44’04.06”). The dwellings and public areas were selected based on the presence of high rat concentrations, as reported by the “Service d’Hygiène de Lyon” (i.e., the department of public health in Lyon). This study formed the basis of a larger project characterizing the distribution of zoonotic pathogens, including *Leptospira*, in rat populations conducted in the Rhône *département* (i.e., administrative sub-unit of France) [24]. For the purposes of this project, trapping locations were chosen based on population density to represent a range of urbanization levels. Only eight locations where *Leptospira* was detected were included in this study because the bacterial load in rats’ kidneys was found to be appropriate for further molecular investigations.

Trapping occurred on both public and private properties, and permission to trap was obtained from the City and the property managers, respectively.

### Sample collection

Previous research has indicated that urban rats form tight colonies with a small home range, suggesting that a colony could be limited to a block [25–26]. Thus, we defined colonies to consist of rats inside geographical barriers, such as rivers, or urban infrastructures, including railways or blocks. Using this definition, the trapping locations were chosen to encompass not more than one colony. The survey was subdivided into a main trapping period, consisting of one 6-month period (October 2011 to March 2012), and a follow-up period, consisting of a 1-week period in January 2013 in which trapping was limited to the “Parc de la Tête d’Or” location. This extra week of trapping was performed to verify the consistency of the former results over time, and the location was chosen based on public health relevance (public garden) and high rat abundance. For the purposes of both pest control and this study, trapping/removal methods were used (snap trapping and live capture), with live capture being the preferred method to prevent post-mortem kidney contamination. Most rats were captured with small (28 cm x 9 cm x 9 cm) or large (50 cm x 15 cm x 15 cm) single-catch rat traps. The captured rats were transported to the laboratory, immediately anesthetized using isoflurane and sacrificed by cervical dislocation. For each animal, the weight, size, sex and level of sexual maturity (*i*.*e*., the presence of seminal vesicles in males and a developed uterus in females) were recorded. The rats that died during capture were frozen at -20°C and thawed on the day of dissection.

One kidney was removed and samples were taken for bacteriological culture immediately following collection, and one fourth of the other kidney, including the cortex and medulla, was stored at -80°C until further analysis.

### Molecular investigations

**Fig 1:**
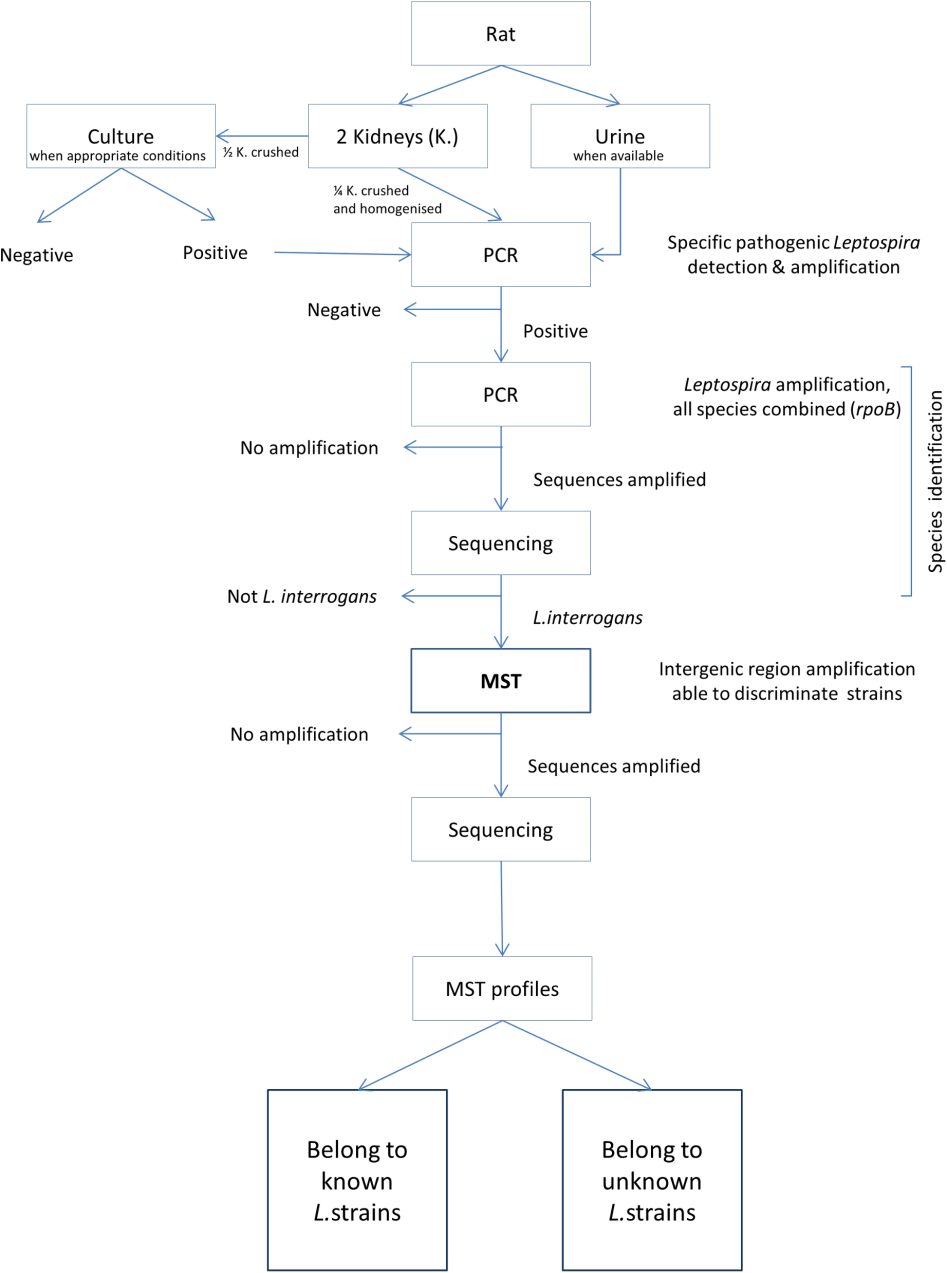
Flow chart of the molecular methods performed to determine *Leptospira* strains.

The flow chart of the molecular investigations is presented in Fig 1 and is detailed as follows.

#### *Leptospira* isolation

Half of one crushed kidney was aseptically transferred to tubes containing Ellinghausen McCullough Johnson and Harris (EMJH) culture media (Indicia, St Génis, France). Three dilution tubes were incubated at 29°C, according to a previously published protocol for the isolation of pathogenic *Leptospira* [27]. The tubes were examined weekly by dark-field microscopy for two months [28]. An additional month of culture examination was performed when PCR analysis yielded positive results (TaqMan real-time PCR kit performed in parallel and detailed below) to increase the probability of detecting *Leptospira* growth.

DNA was extracted from 200 μl of each *Leptospira* culture using a QIAamp DNA Mini Kit (Qiagen, Courtaboeuf, France) following the manufacturer’s instructions. All DNA samples were stored at -20°C.

#### MST profile determination. DNA extraction and *Leptospira* detection in kidney tissue and urine

One fourth of each kidney was homogenized aseptically using a syringe. A small amount of this crushed kidney (ca. 25 mg) or urine when available was incubated with 180 μl T1 Buffer and 25 μl proteinase K for 3 hours. After this protein digestion, DNA was extracted from 200 μl of lysed tissue using a Nucleospin Tissue kit (Macherey Nagel, Hoerd, France) following the manufacturer’s instructions. All DNA samples were stored at -20°C.

The presence of *Leptospira* colonization of the kidney was assessed using a specific pathogenic *Leptospira* TaqMan real-time PCR kit (TaqVet PathoLept Kit, LSI, Lissieu, France) following the manufacturer’s instructions. A final volume of 25 μl, consisting of 20 μl Mix Lepto (LSI) and 5 μl target DNA, was amplified using a Rotor-Gene Q (Qiagen) according to the following program: 10 min of enzyme activation at 95°C, followed by 45 cycles of 95°C for 15 s and 60°C for 1 min. An appropriate negative control, consisting of the PCR mix without the target DNA, was included. The specimens with a cycle threshold (Ct) less than 45 cycles were considered positive.

When available, the DNA extraction and PCR technique was performed on urine samples as well.

#### Molecular typing

The genotyping of *Leptospira* DNA was conducted using extracts from the isolate cultures and from the kidney samples. As the MST that we used was developed to discriminate among strains of the species *Leptospira interrogans*, this species was verified first as follows: the *rpoB* gene was amplified by PCR using HotStarTaq DNA Polymerase (Qiagen) under standard conditions and the primers previously described [29]. A final volume of 50 μl, consisting of 35 μl H_2_O, 5 μl of 10× buffer (Qiagen), 2 μl of 25 mM MgCl_2_, 1 μl of 10 mM dNTPs (Qiagen), 1 μl of forward primer (10 μM), 1 μl of reverse primer (10 μM), 0.3 μl of HotStarTaq DNA Polymerase (Qiagen) and 5 μl of target DNA, was amplified according to the following program: 15 min of enzyme activation at 95°C, followed by 40 cycles consisting of 95°C for 30 s, 56°C for 30 s, and 72°C for 1 min and a final elongation step of 10 min at 72°C. The PCR products were sequenced using the BigDye Terminator sequencing kit and a 3730XL DNA analyzer (Applied Biosystems, Saint Aubin, France). The *Leptospira* species in the samples were identified by analyzing these sequences using the NCBI nucleotide BLAST software (http://blast.ncbi.nlm.nih.gov).

The samples in which the *L*. *interrogans* species was identified were screened using the MST method as reported previously [23]. PCR amplifications were performed in a final volume of 50 μl containing 35 μl of H_2_O, 5 μl of 10× buffer (Qiagen), 2 μl of 25 mM MgCl_2_, 1 μl of 10 mM dNTPs (Qiagen), 1 μl of forward primer (10 μM), 1 μl of reverse primer (10 μM), 0.3 μl of HotStarTaq DNA Polymerase (Qiagen) and 5 μl of target DNA. For samples with a low quantity of *Leptospira* DNA, the volume of the target DNA was increased to 8 μl. The mix was amplified according to the following program: 15 min of enzyme activation at 95°C; followed by 40 cycles consisting of 95°C for 30 s, 50°C or 52°C, depending on the primer, for 30 s, and 72°C for 1 min; and a final elongation step of 10 min at 72°C. PCR mixes without the target DNA and with DNA from *Leptospira interrogans* serovar Copenhageni were used as appropriate negative and positive controls, respectively. The PCR products were sequenced using the BigDye Terminator sequencing kit with an Applied Biosystems 3730XL DNA analyzer (Applied Biosystems, Saint Aubin, France). The *Leptopira* serovars and strains were deduced from a comparison of the sequences obtained with the MST reference sequences using NCBI nucleotide BLAST software (http://blast.ncbi.nlm.nih.gov) and a previously published database [23].

### Spatial analysis

Variations in the locations, infection status and strains of rats trapped across the study area were visualized spatially using the ArcGIS software, version 9.3 (ESRI, Redlands, CA, USA). The mean distance between colonies in which a given strain was identified was compared with the mean distance between colonies in which different strains were identified using Student’s T test; an alpha value of 0.05 was used to identify significant differences. The statistical analyses were conducted using the R software, version 3.0.1 (R Development Core Team (2013), R Foundation for Statistical Computing, Vienna, Austria).

## Results

### *Leptospira* spp. detection

A total of 84 rats were trapped in the eight locations, and all were Norway rats. The distributions of the rats trapped at each site and of PCR-positive samples are displayed in Table 1. *L*. *interrogans* was isolated from 24 rats, and *L*. *interrogans* DNA was extracted from 36 rats’ kidneys. For one rat, a positive culture result was observed together with a negative PCR result. For another rat, *L*. *interrogans* DNA was detected in the urine, whereas it was not in the kidney tissue.

**Table 1:** Distribution of rats testing positive among the trapping locations and nature (species, serogroup [Sg], serovar [Sv] and strain) of *Leptospira* based on multispacer sequence typing profiles.

	No of rats captured	No of PCR^a^-positive kidneys	No of PCR^a^-positive urines (no performed)	No of culture-positive kidneys	Total infected rats	*L*.*interrogans* species (by sequencing)	No of complete MST-profiles	*L*.strains deduced from the MST-profile		
								Sg	Sv	strain
Francheville	9	1		1	1	1	1	IH	cop	M20 or Wijinberg
Guillotière	3	2		0	2	2	2	IH	cop	M20 or Wijinberg
la chapelle	1	0		1	1	1	1	IH	ict	R1
Mermoz sud_B	13	9	2 (3)	9	9	9	9	IH	cop	M20 or Wijinberg
Mermoz sud_F	17	12		6	12	12	11	IH	cop	M20 or Wijinberg
Mermoz sud_R	7	2	1(2)	2	3	3	3	IH	cop	M20 or Wijinberg
PTO	8	5	1(1)	3	5	5	5	IH	ict	CHU Réunion
PTO (2)*	3	2		2	2	2	2	IH	ict	CHU Réunion
UIOM	23	2	0(1)	0	2	1	1	IH	cop	Shibaura 9
Total	84	36	4(7)	24	37	36	35			

^a^ PCR using *rpoB* primer

*second trapping period for the strain followed up in the Parc de la Tête d’Or

IH, Icterohaemorrhagiae; cop, Copenhageni; ict, icterohaemorrhagiae; PTO, “Parc de la Tête d’Or”

### *Leptospira* MST profiles

All 37 rats in which *L*. *interrogans* was detected were typed by MST, and MST profiles were determined in 35 samples; two MST profiles could not be identified due to low DNA concentrations. Four different MST profiles were identified and related to the serogroup Icterohaemorrhagiae and the serovars Icterohaemorrhagiae and Copenhageni (strains are indicated in brackets). The most commonly observed MST-profile-related strain was Copenhageni [Wijinberg or M20] (n = 26), followed by Icterohaemorrhagiae [CHU Réunion] (n = 7), Icterohaemorrhagiae [R1] (n = 1) and Copenhageni [Shibaura 9] (n = 1). All of the MST profiles of the *Leptospira* isolates were similar to the MST profiles of the *Leptospira* DNA from the corresponding kidneys or urine.

The characteristics of the infected-rat populations are displayed in Table 2. All the morphometric characteristics and the laboratory results of the 81 rats collected from October 2011 to March 2012 are available (S1 Table).

**Table 2:** Morphometric characteristics of a population of wild Norway rats with different multispacer sequence typing profiles.

		*Leptospira* strains			
		copenhageni [Wijinberg/M20] (n = 26)	icterohaemorrhagiae [CHU Réunion] (n = 7)	copenhageni [Shibaura 9] (n = 1)	icterohaemorrhagiae [R1] (n = 1)
Characteristic					
sex					
	Female	10	2	0	0
	Male	16	5	1	1
Sexual maturity					
	Immature	1	0	0	0
	mature	25	7	1	1
Weight (g)					
	Median (IQR)	244.3 (204.8–287.0)	387.0 (273.5–431.0)	374 (-^a^)	267 (-^a^)
Lenght (cm)					
	Median (IQR)	22.7 (22.0–23.9)	25 (23.5–26.7)	24.5 (-^a^)	23.3 (-^a^)

^a^ insufficient number of rats to estimate the interquartile range (IQR)

### Spatial patterns observed

**Fig 2:**
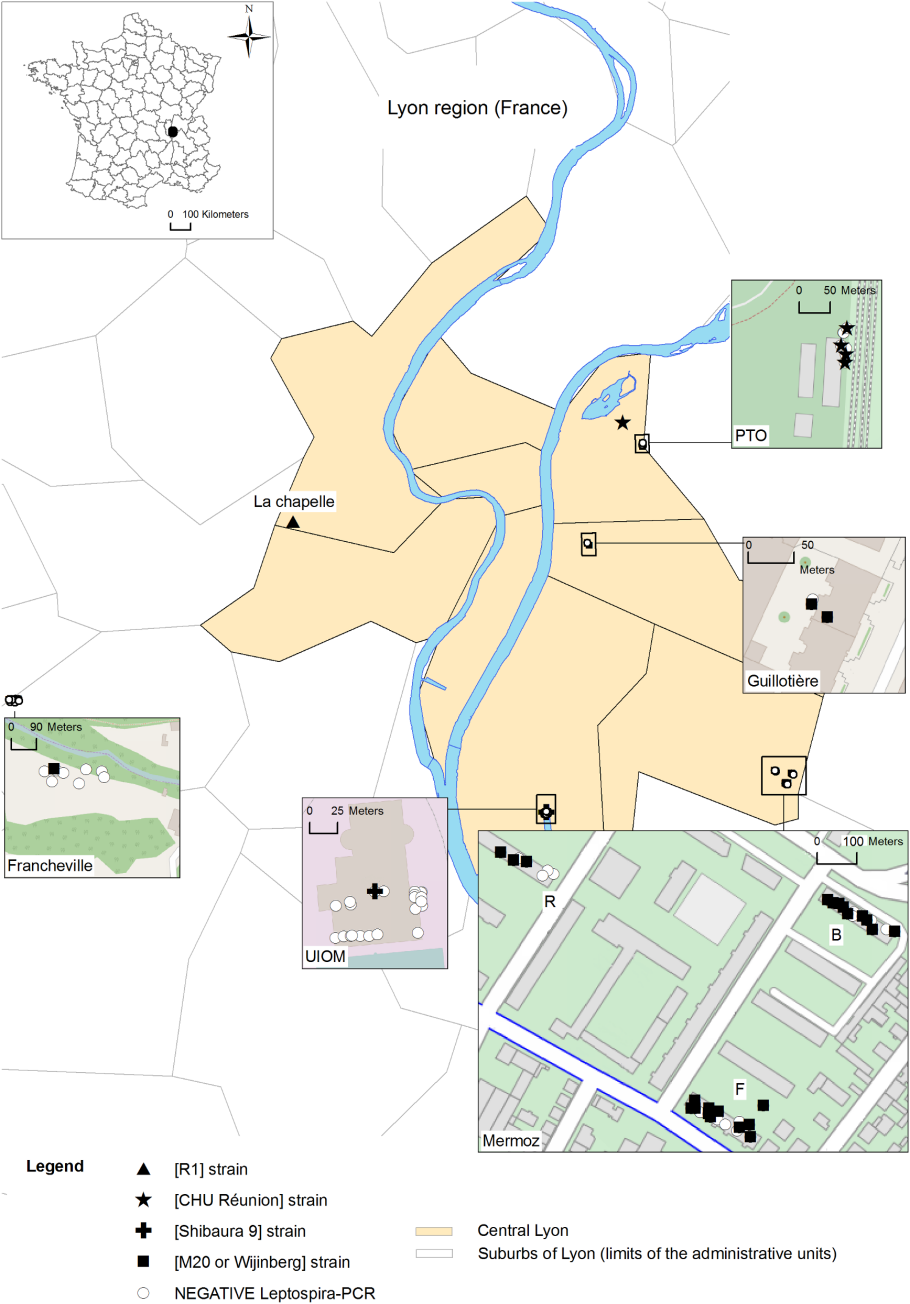
Geographic distribution of *L*. *interrogans*. *L*. *interrogans* strains deduced from MST profiles detected in the kidneys of wild Norway rats from downtown Lyon and a suburb, trapped between October 2011 and March 2012 (contains information from OpenStreetMap®, which is made available here under the CC BY 3.0 License, the original images were modified for representative purposes).

Fig 2 illustrates the distribution of rats infected with *L*. *interrogans* and shows the strains and locations of larger clusters of rats carrying a given strain. Interestingly, one strain was isolated in each colony. The colonies infected with *Leptospira* serovar Copenhageni [Wijinberg or M20] were most common (n = 5); each of the three other strains was found in only one colony.

Among the five colonies infected by Copenhageni [Wijinberg or M20], three locations (“Mermoz-B”, “Mermoz-F” and “Mermoz-R”) were separated by less than 0.5 km. Two other colonies infected by *Leptospira* serovar Copenhageni [Wijinberg or M20] (“Francheville” and “Guillotière”) were farther from the former locations, at 11.5 km and 4.5 km away, respectively. The colony where Icterohaemorrhagiae [CHU Réunion] circulated was located in “Parc de la Tête d’or” and was separated by 2.6 km to 10 km from the other locations. The mean distance between colonies where *Leptospira* Copenhageni [Wijinberg or M20] circulated was 5.5±4.82 km and was not significantly different from the mean distance between colonies that contained different strains, which was 6.88±1.84 km (t = -0.8, df = 10, p = 0.44).

In the two infected rats trapped in the second period in the “Parc de la Tête d’Or”, the serovar Icterohaemorrhagiae [CHU Réunion] was identified from the MST profiles. The serovar detected in this period was the same as the serovar detected in the same location one year previously.

## Discussion

This study has demonstrated that urban rats carry a variety of *L*. *interrogans* strains, all with MST profiles that are concordant with reference strains from the serogroup Icterohaemorrhagiae, which has been previously identified in humans. More specifically, four distinct MST profiles were identified in the rat population in this study, and a single MST profile was attributed to each rat colony. These outcomes raised public health, methodological and epidemiological issues.

### Implications for public health

This study distinguished among four MST profiles related to four strains from the serogroup Icterohaemorrhagiae. As an MST profile is only a partial typing of the entire leptospiral genome, two identical MST profiles are not necessarily evidence of two identical strains; however, two identical MST profiles strongly suggest that the strains are closely related. The MST profiles found in the rats in our survey were identical to those of the reference strains that were isolated from humans in various areas. This finding indicates that the strains circulating in rats and humans are closely related. Assuming that a human patient would be infected in Lyon city center with one of the four MST profiles described in our study, it would be possible to speculate that the transmission occurred via the rat population. Although rats are the main source of *Leptospira* in cities, many reservoir hosts of leptospirosis exist, and to determine whether rats are the source of human disease, an inventory of strains among the varied hosts species and humans is required. In this context, the MST approach could be used for the purpose of source tracing.

MST has previously helped to define phylogeographical lineages of mycobacteria [30]. As the present study suggests that only one strain may be assigned to a colony home range, the areas in which individual strains are distributed are expected to be well delimited without overlapping. Among the numerous colonies that exist in Lyon, the eight colonies in this study provide a restricted overview of currently circulating strains; with more sampling, it would be possible to determine whether additional strains circulate and to provide a more complete picture of their spatial distributions. Maps of the spatial distribution of *Leptospira* strains would be of great interest in epidemiologic investigations. This knowledge would help to locate the potential sources of infection. Together with surveillance of MST outcomes in rats, an updated map of strain distributions would permit early detection of emerging strains. Strain emergence or re-emergence in a given area is likely to occur due to changing environments, host population fluctuations and vaccine pressure. Detecting such a change would facilitate appropriate leptospirosis preparedness efforts and would be useful in preparing for natural disasters, such as storms, hurricanes and particularly flooding, all of which are usually associated with an increased incidence of human and animal leptospirosis [31–32].

Finally, our finding that all of the strains belonged to the serogroup Icterohaemorrhagiae supported the use of the current vaccine available for humans in France, which includes the serovar Icterohaemorrhagiae (Spirolept®). By distinguishing *Leptospira strains*, MST could identify further strains of interest for public health and could aid in the designing of future vaccines.

### Use of MST to distinguish among *Leptospira* strains in rat surveys

MST allows for the direct detection of *Leptospira* in rat tissues; therefore, it is useful in field surveys in which appropriate sampling for bacteriological cultures is limited. The robustness of MST was highlighted by the consistency with which it could identify strains in various samples (*e*.*g*., isolates or *Leptospira* DNA from the kidney or urine) from the same rat. This finding suggested that *Leptospira* DNA extracted from rat samples could be sufficient for MST use.

The MST profiles were not fully obtained for two rats that tested positive for *L*. *interrogans* because the DNA load, characterized by the Ct, was lower in these samples (LYO0845, Ct = 38.67 and LYO0881, Ct = 38.72), compared to other samples (Ct≤35). Because the MST method is less sensitive than real-time PCR, the MST method cannot be used alone for screening purposes. The use of serial testing, consisting of culture and/or qPCR combined with MST, can be expected to provide a reliable reflection of *Leptospira* infection and of the *Leptospira* strains circulating in rat colonies.

The recovery of bacteria from an individual kidney by bacteriological culture was considered to be definitively diagnostic of *L*. *interrogans* infection and was considered to be appropriate for early detection because kidney colonization occurs during the first week of *Leptospira* infection [33]. However, this culturing method is slow and insensitive, and the frequency with which bacteria are isolated is typically low. Thus, qPCR was also performed because it is reportedly the most sensitive method of detection for human *Leptospira* [34]. However, false negatives can still be obtained if PCR is used to identify uncharacterized pathogenic strains [34] or if the bacteria have aggregated in an unsampled part of the kidney. Thus, a combination of culturing and qPCR analyses on a variety of sample types was performed in an attempt to increase detection sensitivity. For instance, one rat that was PCR-positive in the urine and PCR-negative in the kidney would have been missed without this combination of tests.

### Implications for the epidemiology of *Leptospira*

Spatial analysis suggests that a single strain was found in each colony. It cannot be determined whether this specific geographical pattern resulted from the dispersal of individual rats and rat-to-rat transmission or from exposure to a non-rat source, such as the environment within a given colony, or both. As colonies infected with the same strain are not significantly closer to each other than are colonies infected with different strains, a dispersal of individuals resulting in admixed strains would have been expected; however, such a pattern was not observed in this study. Based on the assumption that a single strain was associated with each colony, it is likely that few contacts occur between colonies, which is consistent with previous studies of the genetic characteristics of urban rats [35]. Moreover, high degrees of habitat fragmentation in urban areas should also limit individual rat movement between areas and result in the geographical isolation of urban rat populations [25]. The absence of cross-contamination between colonies strongly supports this hypothesis. Thus, Copenhageni [Wijinberg or M20] infections in rats from remote colonies may be related to rare and ancient rat movements between colonies, as has been described previously in other cities by assessing population genetics [36]. Interestingly, the environment associated with the 5 sites varied considerably (low-income dwelling, old dwellings, public garden in the suburb), suggesting that a variety of habitats may be associated with the same strain.

Although follow-up observations were limited to one location, the fact that the same strain was isolated in the same location one year later suggests that the isolated *Leptospira* strain persist over time within the same colony and supports the hypothesis that there is limited interaction between colonies. Therefore, the strain Copenhageni [Wijinberg or M20] encountered in remote locations may have been there for a substantial amount of time.

One potential method for testing the distributions of strains over space and time would be to tag rats and monitor their movements. Such protocols have been used before in rural or island habitats, although they are less appropriate for urban wild rats, mainly due to the adverse effects of human activities on capture success (e.g., pest control measures and trap deterioration). Thus, our original findings aid in the estimation of temporal stability and spatial connectivity of populations, increasing our knowledge of the epidemiology of this disease, which will be crucial for the development of prevention methods.

## Conclusions

Our study demonstrates the relevance of MST for the accurate characterization of *Leptospira interrogans* extracted-DNA from rat tissues. Improved typing methods are required to investigate the molecular epidemiology of leptospirosis, and MST could aid in achievement of the level of definition that is needed for proper interpretation, thereby offering novel perspectives for epidemiological surveillance and investigations.

In the future, large-scale *Leptospira* genotyping using MST could provide substantial knowledge of leptospirosis in various host populations and habitats. This approach could generate valuable information that could be used to generate a phylogeographical database to improve epidemiological investigations and to track the sources of this disease in a particular epidemiological catchment area. Early detection of emergent strains or changes in the strain distribution using MST could drive future public health efforts.

## Supporting Information

S1 File Supplemental information. ARRIVE check list.
(PDF)

S1 Table Data set. Morphometric characteristics and laboratory results of the 81 rats collected from October 2011 to March 2012.
(XLSX)
